# Effects of Dietary Fiber on Nutrients Utilization and Gut Health of Poultry: A Review of Challenges and Opportunities

**DOI:** 10.3390/ani11010181

**Published:** 2021-01-14

**Authors:** Amit Kumar Singh, Woo Kyun Kim

**Affiliations:** Department of Poultry Science, University of Georgia, Athens, GA 30602, USA; aksingh@uga.edu

**Keywords:** antinutrient, enzyme, fermentation, fiber, gut health, microbiota, meta-analysis, poultry, prebiotic

## Abstract

**Simple Summary:**

The inclusion of agricultural co-products has been increased to utilize the nutrients in these products available at low cost, but inherently, it adds a high dietary fiber content in the poultry diets. The use of exogenous feed enzymes along with advancements in feed milling, feed formulation, and processing of these non-conventional ingredients to improve their digestibility and utilization have played an emphatic role in boosting their use globally. Despite such developments, the presence of a high level of dietary fibers (DF) acting in an anti-nutritive manner still poses challenges in poultry feeding. Various isolated forms of fiber or feed enzymes to break DF into fermentable substrates are being used extensively to provide potential prebiotics to support beneficial gut microbiota or probiotics to improve the gut health of poultry raised without antibiotic growth promoters (AGP). This review reports and discusses the existing challenges in feeding high-DF feed ingredients to poultry and the opportunities that are available to improve the nutritive value of such non-conventional feed ingredients by adopting various technologies.

**Abstract:**

Many fibrous ingredients incorporated in poultry feed to reduce production costs have low digestibility and cause poor growth in poultry. However, all plant-based fibers are not equal, and thus exert variable physiological effects on the birds, including but not limited to, digestibility, growth performance, and microbial fermentation. Several types of fibers, especially oligosaccharides, when supplemented in poultry diets in isolated form, exhibit prebiotic effects by enhancing beneficial gut microbiota, modulating gut immunity, boosting intestinal mucosal health, and increasing the production of short-chain fatty acids (SCFA) in the gut. Recently, poultry producers are also facing the challenge of limiting the use of antibiotic growth promoters (AGP) in poultry feed. In addition to other alternatives in use, exogenous non-starch polysaccharides digesting enzymes (NSPase) and prebiotics are being used to provide substrates to support the gut microbiome. We also conducted a meta-analysis of different studies conducted in similar experimental conditions to evaluate the variability and conclusiveness in effects of NSPase on growth performance of broilers fed fibrous ingredients. This review presents a holistic approach in discussing the existing challenges of incorporating high-fiber ingredients in poultry feed, as well as strategies to fully utilize the potential of such ingredients in improving feed efficiency and gut health of poultry.

## 1. Introduction

The poultry diet is balanced for metabolizable energy and protein through the incorporation of several ingredients and additives. The cost of many cereal grains and legumes used in making poultry feed is increasing due to the growing markets utilizing them as food and fuel [1]. To counter this, alternative feedstuffs such as agricultural co-products, including wheat middlings, soy hulls, oil cakes, distillers dried grains and solubles (DDGS), and sugar beet pulp, etc., are regularly incorporated in poultry feed. However, these co-products inherently contain a high proportion of dietary fiber (DF) comprising of non-starch polysaccharides (NSP), lignin, and other indigestible plant-based carbohydrates [2]. Poultry lacks endogenous enzymes required for the breakdown of these NSP. The NSP fractions include cellulose and non-cellulosic polysaccharides (NCP), and the NCP portion further consists of pectic polysaccharides and hemicellulose [2,3]. DF is further divided into soluble and insoluble fibers based on their aqueous solubility. Furthermore, a non-digestible fraction of starch that remains resistant to enzymatic digestion is termed as ‘resistant starch’ and has been reported to possess physiological functions similar to other DF [4].

Recently, there has been an increase in the trend of incorporating DF and oligosaccharides in poultry diet to supply substrates for beneficial gut microbes [5]. Previous research on the characteristics of fiber demonstrates both opportunities and challenges in enhancing healthy and efficient poultry production. Fiber can act as an antinutrient, as it often encapsulates nutrients in cell walls of plant-based feed ingredients, negatively influences viscosity of digesta, and impacts mineral absorption through the chelating properties of some fiber moieties [6,7]. It has been noted that DF is utilized by microbes in the lower gut to produce short-chain fatty acids (SCFA) as fermentation metabolites [8]. These SCFA are utilized by the intestinal enterocytes for growth and are transported to the liver to produce ATP. However, because of their low digestibility, NSP reduces the apparent metabolizable energy (AME) value of feed, and consequently increases the viscosity of digesta, which adversely affects the digestibility of other nutrients [5]. Thus, preprocessing and enzyme supplementation to increase the digestibility of fiber will also improve utilization of other nutrients in feed and will increase fermentable resources for the gut microbes [5,9,10]. The fermentable substrate can range from complex fragments to simple oligomers that could serve as prebiotics if they could selectively enhance the population of beneficial bacteria leading to immunomodulation and improved gut health [11,12,13].

## 2. Composition and Properties of DF

The generic term ‘fiber’ in nutrition is broadly used for a diverse group of complex carbohydrate fractions of NSP, oligosaccharides and resistant starch, and polyphenolic compound lignin. The proximate analysis system developed by Weende Experiment Station in Germany classified carbohydrates in feed into a more digestible component called nitrogen-free extract (NFE) and a less digestible fibrous component called crude fiber (CF) [14]. The CF is still used in poultry feed formulation and it is determined as the organic residue of acid and alkali digestion, and it fails to account for total fractions of NSP. The other analysis process using neutral and acid detergents by Van Soest [15] categorized fiber into neutral detergent fiber (NDF) comprising of cellulose, hemicellulose and lignin, and acid detergent fiber (ADF), largely consisting of cellulose and lignin. The Van Soest detergent fiber system is also affected by unreliability and falls short of accounting for all NSP in the poultry feed ingredients. The term DF is more associated with the physiological effect and method of determination of fiber component in the feed. The DF in feed is determined either using enzymatic-gravimetric methods adopted by the Association of Official Analytical Chemists for total, soluble, and insoluble DF [16], or by the Upsala and Englyst method that quantifies each monosaccharide converted to aldol acetates and measured using chromatography and spectrophotometry [17,18]. The DF primarily consists of carbohydrates polymers such as cellulose, hemicelluloses, pectins, mucilage, gums, β-glucans, oligosaccharides and resistant starch, and associated substances like lignin [19]. Cellulose is the major component of the plant cell wall and consists of a linear chain of up to 10,000 glucose monomer units per molecule linked by β (1→4) glycosidic bonds. Hemicellulose is a heterogeneous group of chemicals that also include both linear and branched chain of monomers other than glucose. Pectins are gel-forming polysaccharides that are mostly found in the outer skin of rind of fruits and vegetables and consists of polymers of galacturonic acids interspersed with rhamnose and branched chain of pentoses and hexoses. β-glucans are polysaccharides of variable sizes that consist of glucose polymer linked via β-(1→3) and β-(1→6) or via β-(1→4) and β-(1→3) glycosidic bonds. Resistant starch is a homopolysaccharide of glucose that is resistant to digestion by endogenous enzymes and is categorized into various types based on its physical inaccessibility, granular form, retrogradation, and chemical modification.

The physiochemical properties of DF include solubility, water-holding capacity, viscosity and gelation, binding ability, bulking ability, and fermentability [19,20]. Based on the dissolving characteristics of DF, they are either soluble (e.g., pectins and gums) or insoluble (e.g., lignin and cellulose). Notably, DF can hold water in void spaces or hydrophilic sites and the amount of water retained is defined as its water-holding capacity [2]. Viscosity is the property of liquid to resist flow due to internal friction. Viscosity is a proportional relationship between the flow of the fluid and the force directed on it and it relates to DF, where some polysaccharides physically entangle and mix with fluids, thicken, and form gel [21]. Besides, DF can also entrap and bind some bile acids, form bulk due to water holding, and increase fermentation metabolites by being broken down and utilized by the gut microbes. In poultry feeding, soluble DF is desired for enhanced action of gut microbes but there is also an increase of the unstirred water layer on the intestinal mucosa in case of viscous fiber that decreases the efficiency of nutrient absorption.

## 3. Antinutritive Effect of DF in Poultry

DF from different cereals such as wheat, rye, and barley, etc., either in insoluble or soluble forms, can exert an antinutritive effect in poultry by depressing AME, starch digestibility, nitrogen retention, and other nutrient utilization, leading to poor growth performance [7]. Despite the positive attributes of DF, the inclusion of a high level of fiber is limited either because certain NSP can bind bile acids, fats, or cholesterol, and cause lipid malabsorption, leading to low AME value of feed and poor growth [22,23,24].

The viscosity of digesta is one of the major factors impacting digestibility. It is thought that higher viscosity interferes with efficient nutrient diffusion, subsequently reducing their breakdown and transport by endogenous enzymes at the mucosal surface [6,25]. The antinutritive effect of pentosans such as arabinoxylans and arabinogalactan also depends on the degree of polymerization, which in turn increases their viscosity [7]. When intact arabinoxylans (30 g/kg) and depolymerized arabinoxylans (30 g/kg) were added to the broiler diet in Choct and Annison’s [7] study, the ileal viscosity compared with water increased from 1.2 in the control to 2.2 in the depolymerized arabinoxylan, and it increased to 3.0 in the intact arabinoxylan-added diets. In the same study, it was found that when 35 g/kg of arabinoxylan was added to the diet, the digesta viscosity increased by more than two times. It has been reported that the addition of soluble NSP such as arabinoxylan in broiler diet can increase the endogenous loss of amino acids and depress the ileal digestibility of protein [3]. In a study on broiler chicken conducted by Kluth and Rodehutscord [26], it was found that inevitable endogenous loss of CP in low-fiber (CF = 30 g/kg) diet was 11.7 g/kg dry matter intake (DMI), while in the high-fiber (CF = 80 g/kg) group it was 16.3 g/kg. This loss for lysine and methionine was 0.4 and 0.17 g/kg respectively, in low-fiber diet and 0.59 and 0.19 g/kg respectively, in high-fiber diet. Angkanaporn et al. [27] found that adding 15 g/kg of a wheat pentosan (arabinoxylan) decreased the average apparent amino acid digestibility by 17% and increased the average endogenous amino acid loss by 23.5 g/kg DMI. This provides a relatively favorable environment for the establishment of fermentative microbiota in the upper gut, which may not typically reside there in high numbers [6,7]. Moreover, fermentation occurring at the site of the upper gut is not beneficial for the host, as it yields relatively low amounts of energy compared to typical enzymatic digestion and nutrient absorption by the host [28]. Jørgensen et al. [29] reported that NSP fermentation could only contribute up to 3–4% energy of ME intake. However, soluble NSP is more easily digested than insoluble NSP and some of these soluble fibers, such as inulin and wheat dextrin, would not reduce the digestibility of other nutrients, as they do not increase the viscosity of digesta [22]. When a wheat and barley-based diet was supplemented with 7.5 g inulin by replacing an equal amount of wheat in a 28-day broiler study by Rodriguez et al. [30], the viscosity of the jejunal digesta reduced from 1.83 in control to 1.30 millipascal seconds (mPa s). If the viscosity of digesta is managed by using feed additives, then soluble fibers can be better utilized by poultry due to reduced interference of the movement of the digesta and improved diffusion of digestive enzymes to the substrates [7].

The insoluble fiber present in poultry feed causes less viscosity than soluble fiber and has low fermentability due to its limited accessibility by the action of microbial or host enzymes. The insoluble fiber binds water by surface tension or hydrogen bonds in the pores of its matrix, and the quantity of water it can bind depends upon its swelling characteristics or water-holding capacity [2]. As such, a poultry diet containing a higher amount of insoluble fiber can increase the transit rate of digesta and passage of nutrients in the lower gastrointestinal tract due to this higher water-holding capacity [19]. However, in poultry, it is reported that coarse particles can delay the transit of digesta in the gizzard and thus increase the exposure of substrates to the digestive enzymes [31]. Some authors suggest that it is not the water-holding capacity of insoluble DF, but rather it is the mechanical stimulation, that leads to excess mucus secretion and increased peristalsis in response to coarse particles that increase motility and decrease digesta transit or retention time [32,33]. In a pig study by Wilfart et al. [34], it was found that the addition of around 0.8% insoluble fiber in the diet reduced the mean retention time of digesta in the total tract by 9 h when the solid-phase marker was used. Moreover, encapsulation of other nutrients by fiber in the cell wall of plant-based feed also reduces the utilization of nutrients and limits their digestibility in several feedstuffs [5,35]. Downstream consequences of high NSP feed ingredients with high water retention properties, such as wheat, barley, oats, cassava, and rye, include wet droppings and increased moisture content of litter. In turn, these conditions lead to poor foot-pad quality and increased ammonia volatilization [36,37]. Together, these data suggest that high NSP feed ingredients may adversely affect poultry health both directly and indirectly.

It is not well-defined how DF would decrease the bioavailability of minerals and vitamins, but the adsorption property of DF is expected to reduce the utilization of these nutrients by the host. The presence of higher levels of phytate associated with fiber increases the excretion of endogenous minerals in broilers [38]. Cowieson et al. [38] reported that in a precision feeding assay on 6-week-old female broilers, feeding of 1 g phytic acid increased endogenous excretion of calcium by 69%, iron by 31%, sodium by 300%, and sulfur by 47%. It is established that phytate present in most plant-based fibers can strongly bind phosphorus and divalent cations such as zinc, copper, calcium, and magnesium, thus reducing their absorption and disturbing their homeostasis in the body [39,40].

## 4. The Beneficial Effect of DF in Poultry Nutrition and Gut Health

### 4.1. Effects of Fibrous Diet on Nutrient Utilization and Ammonia Emission

The dietary protein and amino acids that escape host digestion are subject to fermentation by gut microbes. Approximately half of these nitrogen sources are metabolized to uric acid and ammonia in the gut, thus depriving nourishment and increasing toxicity in the host [41]. The uric acid is subsequently volatilized to ammonia in the litter by the microbes, which causes respiratory discomfort to the birds and poses a major public health concern [42]. Although carbohydrates are the preferred substrate for energy metabolism by the gut microbes, depletion of carbohydrate substrates causes specific groups of putrefactive and proteolytic microbes to turn to residual protein breakdown and shift fermentation from saccharolytic to proteolytic [43,44]. Besides emitting odorous sulfur compounds and ammonia, fermentation of protein also yields other harmful metabolites such as amines, phenols, and indoles [45]. During the microbial fermentation of DF, nitrogen sources such as ammonia are also utilized for bacterial protein synthesis, which may reduce its emission [46]. It follows that some reports suggest that the inclusion of a higher amount of fermentable DF, combined with the reduction in crude protein in the diet of chicken, has been reported to reduce ammonia emission [47]. The authors stated that the addition of 10% corn-DDGS in the corn-SBM control diet, decreased 7-day cumulative manure ammonia emission from 3.9 g/kg of manure DM to 1.9 g/kg of manure DM, a reduction by 51%. It can be summarized that fermentable fiber provides energy for microbial protein synthesis and prevents the fermentation of undigested protein into ammonia.

### 4.2. Poultry Gut Microbiome and Its Modulation by DF

The gut microbiome is being regarded as an essential and integral part of the gastrointestinal tract (GIT) ecosystem, which functions as an additional organ and contributes to various aspects ranging from nutrient utilization to improved health status and immune modulation in the host [48,49,50,51]. The GIT of poultry is the shelter for a diverse community of microorganisms which comprises over 900 species of bacteria, along with some protozoa, fungi, yeast, and viruses, collectively referred to as microbiome or microbiota that assist the host in breakdown and utilization of consumed feeds [52,53]. The microbiome is present throughout the GIT of poultry from crop to colon, with their population increasing gradually along the distal intestine, and the vast majority reside in the caecum and colon, ranging from 10^11^ to 10^12^ colony forming unit (CFU)/g of luminal content [52]. Various bacterial species reside in different microhabitats of the GIT, ranging from the lumen to mucus and mucosal linings, and are found in significant quantity and diversity [54,55]. A normal process of mucus secretion, epithelial turnover, and peristaltic movements occurring in the GIT is expected to distribute the subsets of the luminal microbiome to the mucus and mucosal surfaces [50]. Rinttilä and Apajalahti [56] reviewed that the GIT environment in chicks is more aerobic initially and is first colonized by facultative aerobic bacteria, such as *Enterobacteriaceae*, *Lactobacillus*, and *Streptococcus*. Later, the GIT gradually transitions to anaerobic, subsequently inducing outgrowth of obligate anaerobes in the growing chicks. The lower gut microbiome depends on the residual digesta and intestinal secretions for deriving nutrients and energy for their growth [57]. The normal microbiome of the lower gut does not compete with the host for nutrients as they utilize the residual feed, salvaging a considerable proportion of energy for the host through fermentation, and precluding colonization of pathogenic and putrefactive bacteria [50]. The composition of this microbiome initially depends on the inoculum passed from the breeder hen as well as the surrounding environmental condition for the chicks during hatch, and later gets modified with age, diet type, and the intestinal environment of the birds [51,52,55,58]. Thus, with the growth of birds, diet serves as one of the strongest determinants of microbial diversity and colonization in the gut.

The bacterial population is the component of the microbiome of major interest for poultry nutritionists because of its role in fermentation and being a target of various AGPs activity. In the ceca of chicken, many families of bacteria, including *Lachnospiraceae*, *Ruminococcaceae*, and *Veillonellaceae*, belonging to the order *Clostridiales*, are non-pathogenic, produce SCFA (lactate, propionate, and butyrate), and are characteristically different than pathogenic *Clostridium perfringens* [56]. In addition to the normal gut microbiome in poultry, several probiotic strains are also added in the poultry feed to enhance the population of known beneficial microbes to prevent dysbiosis or to limit the use of AGP typically implemented to reduce the load of pathogenic microbes. In general, the beneficial bacteria are associated with the promotion of gut maturation and integrity, modulation of the immune response of the host, and persistent antagonism against the pathogen colonization in the gut [49]. However, for practical use as probiotics, beneficial bacteria should fulfill specific criteria: possess antimicrobial activity, adhere to the mucosal lining, have phenotypic and genotypic stability, resist lysosomal destruction and AGP in feed, tolerate acid and bile, and utilize carbohydrates [50].

The inclusion of fermentable DF in the poultry feed supports the growth and establishment of beneficial microbes and probiotic bacteria by providing them substrates for extracting energy and fueling their metabolism. As discussed previously, all DF included in feed are not the same, and those providing benefits to the host by selectively stimulating the growth of beneficial and commensal bacteria in the gut are defined as prebiotics [59]. However, some prebiotics can directly stimulate the immune system and bind the pathogen to facilitate their removal [60,61]. The pathogen could bind with the feed oligosaccharides, mimicking host cell receptors instead of adhering to the host cell surface oligosaccharides, and ultimately get flushed out of the GIT. For example, galactooligosaccharides (GOS) have been found to prevent adhesion of enteropathogenic *Escherichia coli* (EPEC) in human intestinal cells in cell culture and mannan oligosaccharides (MOS) has been reported to decrease the population of *Salmonella* in broiler chicks [61,62]. In general, the most common prebiotics are small fragments of carbohydrates which are oligosaccharides of fructose, xylose, mannose, or galactose, etc., although inulin, raffinose, and resistant starch are used as well [8,11]. The potential of oligosaccharides to modify the intestinal microbiota in poultry is dose-dependent [63], and it has been reported in some studies that oligosaccharides or different fibers with a lower degree of polymerization are more thoroughly broken down through fermentation [12]. Inclusion of DF in poultry diet can effectively support cellulolytic and beneficial bacteria, including *Lactobacillus* and *Bifidobacterium*, and enhance the production of SCFA, and a combined effect would prevent digestive disturbances and wet litter [23,50,64]. Beneficial bacteria present as a part of the gut microbiome also produce metabolites such as bacteriocins that provide protective activity against pathogenic bacteria in poultry [50,54]. Compared with the probiotics added in poultry feed, ingredients containing fiber with potential prebiotic effects provide the advantage of stimulating such commensal and beneficial microbes that are normally present in the host GIT [65]. The degradation of fiber in high-fiber diets could also enhance the population of fiber fermenting microbes, including bacteria of genus *Lactobacillus*, family *Ruminococcoceae*, and family *Lachnospiraceae* [66]. Thus, the fermentable DF can modulate the gut microbiome and promote the growth of beneficial bacteria that would be required to improve broiler performance in the absence of AGP in the diet. However, more research is required to understand the interaction of different components of DF with microbes in a dynamic and competitive gut environment.

### 4.3. Microbial Fermentation of DF

The principal metabolites of DF fermentation are SCFA, which mainly include acetate, propionate, butyrate, lactate and succinate, H_2_O, and gases (CO_2_, H_2_, and CH_4_), along with the accumulation of bacterial cell biomass [25]. The fermentable carbohydrates of hexose and pentose sugar monomers are converted to pyruvate through glycolysis and the pentose phosphate pathway, which can then be converted to lactate, propionate (via succinate), acetate, and butyrate (via acetyl-Co-A) [57]. Acetate is the major SCFA produced in poultry GIT, followed by either propionate or butyrate based on the diet type and site of the GIT [66,67,68]. The production of SCFA depends on the availability of the fermentable substrates, and a high-fiber diet does not always increase SCFA, as Walugembe et al. [69] reported that reducing NDF from 15% to 10% increased cecal butyrate production by 37% on average in both broiler and layer birds. In addition, other SCFAs including valerate, isobutyrate, and isovalerate are produced in trace amounts in the poultry GIT [66,67]. Besides SCFA, lactic acid is also produced in significant quantities, with the highest concentration in the ileum, followed by the crop, gizzard, and ceca [68]. It has been reported that 95–99% of the total SCFA produced in the gut is rapidly absorbed in the gut lumen before it can reach the rectum of non-ruminant animals [4,25]. The ceca in poultry serve as the major site for fermentation and production of SCFA and methane [70]. The SCFA produced can provide up to 5% to 15% of daily metabolizable energy for the maintenance energy requirement of birds [56].

It has also been noted that probiotic bacteria such as *Lactobacillus* increase the production of butyrate in chicken, which has been attributed to the fact that there may be cross-feeding of lactate to the butyrate-producing bacteria [71]. The proportion of production of different SCFA compared to the total production varies based on the type of ingredients and the microbiota dynamics in the gut [44,72]. The dietary feeding of *Lactobacillus plantarum B1* in the finisher phase and during the total period of 6 weeks in broilers has been noticed by Peng et al. [73] to increase the production of propionate by more than 27.5% and total SCFA by more than 30.5% compared with the control diet. In broilers fed the diet supplemented with 16,000 birchwood xylan unit (BXU)/kg of xylanase enzyme for 42 days, Lee et al. [74] observed that the enzyme increased the cecal acetate, propionate, and butyrate by more than 20%, 30%, and 40% respectively, while it decreased the production of branched-chain SCFA by a small amount. Rehman et al. [75] reported that supplementing broilers’ diet with 1% inulin did not influence total cecal SCFA at day 42 but increased the proportion of butyrate to 15.6% from 11.7% in the control. More favorably, the supply of DF is essential in maintaining saccharolytic fermentation and it can influence intestinal physiology indirectly through its metabolic products.

### 4.4. Role of SCFA on Gut Health of Poultry

In addition to supplying energy, SCFA also contributes to the normal functioning of the lower GIT by acting on the intestinal musculature and vasculature and through their impact on the metabolism of enterocytes and colonocytes [76,77]. Among all SCFA, butyrate has received the most attention due to its nutritional properties for intestinal epithelial cells and its inhibitory effect on pathogenic bacteria in the gut. The presence of SCFA in the GIT can affect both gut motility and ionic absorption [76]. During a surgical catheterization experiment in dogs, it was observed that acetic acid was a more potent stimulant for ileal motility based on propulsive motor events, while propionic acid was less effective and butyric acid tended to decrease ileal contraction [78]. In an in vitro study on rat colon by Binder and Mehta [79], it was concluded that stimulation of Na and Cl absorption was greater in response to mucosal butyrate than that of propionate and acetate. The enteral nutrition of SCFA, including acetate, but preferentially butyrate and propionate, could lead to cecal crypt proliferation and act as luminal trophic factors on the cecal epithelium. Butyrate is the preferred source of energy for the enterocyte, where it is readily absorbed via passive diffusion and recognized to regulate the differentiation and proliferation of these cells [80]. In addition to supporting the growth of villi, butyrate can also suppress the invasion of epithelial cells by pathogens [81]. Fernández-Rubio et al. [81] reported that feeding of 0.92 g/kg sodium butyrate as a supplement in a standard broiler diet to orally challenged chicks reduced *Salmonella* Enteritidis at day 42 in the ceca (>10^8^ CFU/g in 8 birds in control vs. >10^8^ CFU/g in 1 bird in sodium butyrate group) and crops (10^7^–10^8^ CFU/g in 12 birds in control vs. 10^7^–10^8^ CFU/g in 2 birds in sodium butyrate group). Butyric acid fed to chickens in the form of impregnated microbeads in the feed in a study by Van Immerseel et al. [82] significantly reduced the colonization of *Salmonella* Enteritidis in the caecum but not in the spleen and liver.

Birds vaccinated against coccidiosis and receiving butyric acid can obtain additional benefits of maintenance of intestinal villi structures and better performance when challenged with coccidiosis [83]. The authors in the study of Leeson et al. [83] observed that villi to crypt ratio increased from 5.3 to 5.9 and final week weight gain increased by 25% when 0.2% butyric acid was supplemented in the diet of the birds challenged with coccidia. It has been understood that SCFA reduces intestinal pH and limits the growth of acid-sensitive pathogenic bacteria like *Enterobacteriaceae* by exhausting their H^+^ATPase pump. This occurs as SCFA overcome the proton motive force across the bacterial cell membrane, enter the cell in undissociated form, and cause damage by dissociating in the neutral cytoplasm of the bacterial cells [84]. Moreover, with increased production of SCFA, pH is reduced, which causes ionization of ammonia and reduces its absorption in the hind gut [43,44,45]. Therefore, it can be inferred that production of SCFA by lower gut microbes does not only salvage the energy from the undigested nutrients, but it also improves the intestinal health of poultry.

### 4.5. Effect of DF on the Gut Histomorphometry, Integrity, and Immune Response

The epithelial cells of the mucosa that are responsible for the absorption of nutrients exist in a dynamic state, dying and shedding regularly, and quickly replenished by the new cells generated from crypts. The area and length of the villi, the depth of the crypts, and the ratio of the villi to the crypts provide a measure of absorption efficiency and gut health status [64,85]. The effect of DF on mucosal morphology in poultry is not well-established, but it has been known to affect cell turnover based on its physicochemical characteristics and inclusion level in the diet of birds in different growth phases [6,64]. The effect of fiber on intestinal histomorphological status is variable: a reduction in villi height has been reported in chickens fed viscous ingredients such as citrus pectin and gum xanthan, while insoluble fiber has been reported to favor villi development [6,25]. In contrast, Andoh et al. [86] observed that the ingestion of pectin increased both villus height and crypt depth in rats. Inclusion of lignin and MOS in the diet of broiler chicken can potentially increase villus height and goblet cell number and thereby may enhance feed efficiency [64]. When MOS was included in the diet at 0.2% until day 21 and 0.1% from day 21 to day 42 in the study by Baurhoo et al. [64], it increased the number of goblet cells per villus from 61 in control to 118 in the jejunum of broilers at day 42. Supplementation of 0.5% fructooligosaccharides (FOS) in broiler diets for a 21 day study by Shang et al. [87] revealed that FOS significantly increased villi height by 24% and mucosa thickness by 26% in ileum compared with control. An increase of 134% in duodenal villi height was also discovered by Ashraf et al. [88] in heat-stressed birds when fed 0.5% MOS prebiotics. The addition of MOS has been reported to increase villi height and goblet cell number, as well as enhance gut integrity in chickens [64]. Thus, the feeding of specific components of DF may stimulate GIT mucosa to increase villi length and surface area for better nutrient absorption, leading to a subsequent higher growth rate in chickens [6]. DF, or its degraded fragments that increase SCFA, especially butyrate, are expected to increase villus height/crypt depth ratio and improve the absorptive capacity of the intestine [4]. Importantly, the enhanced digestive and absorptive performance in response to the increased surface area of villi is associated with increased production of brush border enzymes and higher availability of nutrient transporters [89], thus providing further capacity for nutrient uptake.

Besides their contribution in nutrient utilization in poultry, intestinal epithelium and its mucus secretion also play an important role in defense against pathogens. The epithelial cells are connected by various junctional complex that consists of tight junctions, adherens junctions, gap junction, and desmosomes. The tight junction proteins block the paracellular pathway and regulate intestinal permeability, while claudin, occludin, and junctional adhesion molecule (JAM) family are other crucial transmembrane proteins that associate with peripheral scaffolding proteins, such as ZO family that anchors strands to the actin component of epithelial cells [90,91]. The abnormal changes triggered by pathogens can impair the functions of these tight junction proteins during inflammation and cause increased intestinal leakage [92]. It is also interesting to note that the addition of xylanase enzyme to the wheat-based diet increases expression of mRNA of the tight junction gene occludin, in the ileum of chickens with mucosal barrier impaired by *Clostridium perfringens* [93]. It is also plausible to state that the production of butyrate by fermentation of fiber can enhance intestinal epithelial barrier function via upregulation of tight junction protein claudin-1, and induce ZO-1 and occludin redistribution [77,94]. The increase in abundance of beneficial bacteria such as *Lactobacillus* by prebiotics can also play an important role in regulating intestinal tight junction protein and enhance epithelial barrier function [95]. The proper regulation of tight junctions is important as it can also be affected by immune cells such as tumor necrosis factor (TNF) and interferon gamma (IFNγ) and dysregulation in mucosal immune homeostasis can lead to barrier dysfunction and onset to other diseases [96].

The digesta matrix containing the nutrients from the feed are in intimate contact with the immune system in the gut (gut-associated lymphoid tissue, GALT), which is necessary for proper functioning and development of the immune components and antigen-presenting cells [13,97,98]. Compared to other specific nutrients, the impact of DF on immunity is less explored. It has been suggested in several studies that the bacteria and their components can stimulate and activate immune cells of GALT [48,49,60], suggesting that aspects of DF likely influence immunity by proxy. More directly, SCFA such as butyrate that is produced during fermentation can increase the activity of phagocytic cells and spare glutamine to be used by lymphocytes as a source of energy [13]. Rezaei et al. [10] observed that feeding of 0.5% and 1.0% oligosaccharide extract from palm kernel expeller and coconut flour increased immunoglobulin A (IgA) by 85% and 141% respectively, in 3-week broilers, which could provide some protection against pathogens such as *Salmonella*. FOS supplementation in hen diets significantly enhanced IgA secretion and Toll-like receptor-4 in the intestine and reduced *Salmonella* colonization in the ceca of laying hens [9]. Furthermore, the inclusion of 0.5% FOS in the diet of chicken could also increase IgM and IgG titers in the plasma [60]. Dietary supplementation of the yeast cell wall that is rich in β-glucan has also been found to increase mucosal IgA secretion, increase humoral as well as cell-mediated immunity, and potentially acts as an adjuvant to enhance the immune response against coccidiosis [99]. It is also noteworthy to mention that inclusion of DDGS that contains a high level of NSP and yeast β-glucan has been described to increase IgA, IgG, and gene expression of IL-4 and IL-6 in broilers [100]. In another study on broilers, it was observed that feeding of equal proportions of sugar beet pulp along with rice hulls at a 3% inclusion increased antibody titer against Newcastle disease virus by 100% [101]. The supplementation of oligosaccharides has also been reported to reduce the count of heterophils in chicken, which is suggestive of its stress-relieving action [10,54]. Stress in poultry is also known to adversely affect epithelial integrity, gut permeability, and immune response, and has been reviewed and studied elsewhere [102,103]. Thus, the inclusion of fermentable DF in the diet of poultry could enhance mucosal health, improve immune regulation, and modify the luminal environment for better absorption of nutrients.

## 5. Effects of Enzymatic Degradation and Processing on the Utilization of DF

The lack of information about the chemical composition of DF incorporated in several studies makes it difficult to compare the effects of physiochemical properties of these DFs on its nutritional value in poultry diet. Additionally, the poultry diet contains variable ingredients that are expected to result in differences in the enzymatic digestion and microbial fermentation of DF in different feed matrix. Exogenous feed enzymes can reduce the bacterial colonization in the ileum by reducing the nutrients available for fermentation [68,104]. Feed enzymes can provide benefits to the birds by releasing more nutrients for utilization by the host while providing degraded products such as oligomers of polysaccharide substrates for utilization by the cecal microbes for the production of SCFA [105]. Multi-carbohydrase enzyme supplementation in a wheat-based diet improves nutrient utilization, reduces digesta viscosity, and mitigates the negative impact of *Clostridium perfringens* challenge in broilers [104]. de Vries et al. [106] reviewed that the processing of fibrous feed ingredients by hammer and roller milling can increase the solubility of NSP-fraction and enhance the coefficient of digestibility in poultry. The authors also stated based on several studies that the application of feed enzyme to the ingredients subjected to hydrothermal processing can increase the digestibility of fiber fraction up to 1.5–6 times compared with that of unprocessed diets. There are limited processing techniques in use in poultry feed production to improve the utilization of DF but pelleting and micronizing have been reported to increase the action of pentosanase on fibrous diet [107]. Exogenous NSPase, phytase, and xylanase can increase the bioavailability of several nutrients affected by high-fiber content in feed and concurrently provide degraded fiber fragments and oligosaccharides for utilization by the gut microbiome [5,105]. These NSPase enzymes can decrease digesta viscosity and alleviate the deleterious effect of viscous fiber on the intestinal mucosa in poultry [35]. The use of such exogenous enzymes is thus an efficient method for removing the nutrient encapsulating effect of plant cell walls and generating biologically active oligomers for sustaining the gut microbiome and limiting the use of AGPs for maintaining gut health of poultry. With existing challenges of the antinutritional effect of DF, further research is warranted to explore the opportunities of enhancing the utilization of agricultural co-products through but not limited to chemical, enzymatic, irradiation, and milling techniques.

## 6. Scope of Improvement in Poultry Productivity by Exogenous Fiber-Degrading Enzymes

### 6.1. Meta-Analysis of the Effect of NSPase on Growth Performance of Broilers Fed Fibrous Diet

We summarized the effect of NSPase enzymes on average daily gain (ADG) and feed conversion ratio (FCR) of commercial broilers fed wheat, rye, and barley-based fibrous diet. To identify relevant studies, we searched the web of science core collection and google scholar for literature in accordance with the Preferred Reporting Items for Systematic Reviews and Meta-Analyses (PRISMA) 2009 checklist and population, intervention, comparison, outcome, and study type (PICOS) based search strategy. The search was targeted within the title, abstract, and indexed keywords using the following terms: xylanase or enzyme *, chicken * or broiler *, growth or performance, fiber or fibre, or NSP or polysaccharides. After combining all search results within two decades and removing duplicates, the collection was narrowed to 125 research articles. From this collection, we set exclusion criteria as protease enzyme use and in vitro trials, and inclusion criteria as randomized animal study with wheat, barley, and rye as fiber sources. We filtered 35 eligible studies involving broilers and analyzed 17 studies with comparable trial duration ranging from 20 to 25 days, while the longer and shorter period studies were excluded. Data from control and treatment groups were extracted and the effect size was calculated as the difference in means (Table 1). All the parameters included in the meta-analysis were on the same scale (unit of measure) and had the same outcome (continuous), so the standardized mean difference estimates (Cohen’s d or Hedges’ g) were not used. Review Manager v5.4, RevMan [108], was used for statistical analysis and generation of forest plots using random-effects model to accommodate for higher heterogeneity among studies.

**Figure 1:**
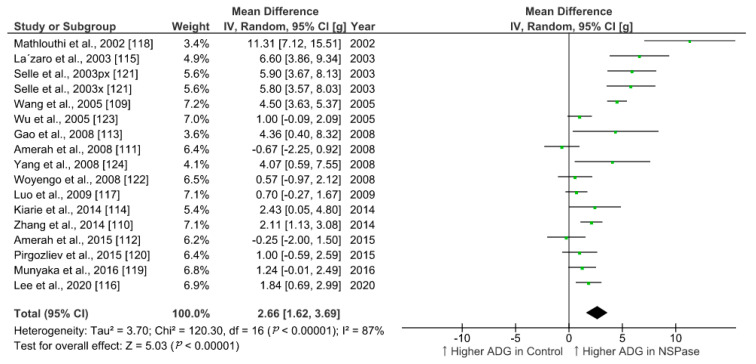
Forest plot of comparison: Effect of non-starch polysaccharides-degrading enzymes on average daily gain (g/day) of broilers fed either wheat or barley or rye-based diets during a trial duration of 20–25 days. Abbreviations: ADG: average daily gain; px: phytase + xylanase; x: xylanase; IV: inverse-variance; CI: confidence interval; Tau^2^: tau-squared; Chi^2^: chi-squared; df: degrees of freedom; I^2^: I-squared statistic; Z: Z-test statistic. The vertical line corresponding to the value of zero (0) in the plot is the line of no effect. The hyphen (-) represents a negative mean difference (effect size) and corresponds to a higher average daily gain in control compared with NSPase group. The squared green boxes represent the point estimates and the width of the horizontal lines extending from the squared green boxes represent the 95% confidence interval of the individual study. The mid-point of the green boxes represents the mean effect estimate and the area of the boxes represent the corresponding weight given to each study. The diamond at the bottom of the studies represents the summary estimate and confidence interval of all studies combined. The points on the vertical axis of the diamond represents the overall effect and the width of the diamond represents the 95% confidence interval. The upward arrow in the label of the plot visually represents a higher average daily gain.

**Figure 2:**
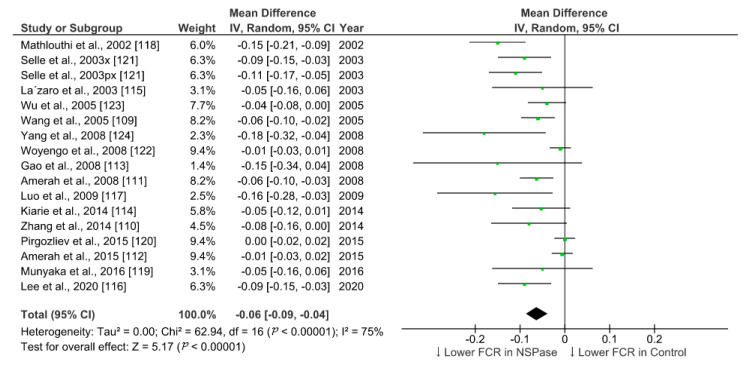
Forest plot of comparison: Effect of non-starch polysaccharides degrading enzymes on feed conversion ratio in broilers fed either wheat or barley or rye-based diets during a trial duration of 20–25 days. Abbreviations: FCR: feed conversion ratio; px: phytase + xylanase; x: xylanase; IV: inverse-variance; CI: confidence interval; Tau^2^: tau-squared; Chi^2^: chi-squared; df: degrees of freedom; I^2^: I-squared statistic; Z: Z-test statistic. The vertical line corresponding to the value of zero (0) in the plot is the line of no effect. The hyphen (-) represents a negative mean difference (effect size) and corresponds to a lower feed conversion ratio in NSPase group compared with control. The squared green boxes represent the point estimates and the width of the horizontal lines extending from the squared green boxes represent the 95% confidence interval of the individual study. The mid-point of the green boxes represents the mean effect estimate and the area of the boxes represent the corresponding weight given to each study. The diamond at the bottom of the studies represents the summary estimate and confidence interval of all studies combined. The points on the vertical axis of the diamond represents the overall effect and the width of the diamond represents the 95% confidence interval. The downward arrow in the label of the plot visually represents a lower feed conversion ratio.

In all the studies analyzed, xylanase was used as NSPase enzyme either alone or in combination with other carbohydrases. Few studies evaluated the effect of xylanase in conjunction with phytase included in the basal diet. Overall, the supplementation of NSPase enzyme seems to improve ADG in broilers from 20 to 25-days of age by 2.5 g/day and decrease FCR by 6 points compared to fibrous control diet. Compared to more recent studies, research outcomes from previous decades show higher effect sizes for ADG in response to enzyme treatments (Figure 1). This trend was not obvious in case of summary of effects on FCR (Figure 2). It is understandable that ample progress has been made in birds management and feed manufacturing technology which could have helped birds to grow better in recent times [5,106]. There might be a limit on improvement in performance in response to NSPase enzymes when birds are already able to perform to their potential. However, the effectiveness of such exogenous feed enzymes and their combination should also be evaluated in terms of gut health improvement, which would be more detectable under challenged and unhygienic rearing conditions [5,105]. Xylanase is just one among the other NSPase enzymes such as glucanase, amylase, phytase, cellulase, and mannanase, etc. Data from more studies need to be compared for conclusive interpretation on the effect of enzyme combination and types of ingredients used in poultry feed. Moreover, there are other various parameters such as carcass yield, meat quality, litter quality, and digestibility, etc., that would also need to be considered apart from the enzyme activity in feed for evaluating the efficiency of NSPase enzymes. Nonetheless, the meta-analysis of the effect of xylanase on ADG and FCR provides a quantitative assessment of its efficacy across different studies where broilers were fed a fibrous diet. The improvement in growth performance of broilers in response to exogenous enzymes is based on underlying mechanism of improvement in the digestibility of nutrients [109,110]. Thus, further statistical analysis of the results of multiple studies are required to ascertain the effect of NSPase enzymes on the ileal digestibility of the components of NSP that corresponds to the improvement in the growth of broilers.

**Table 1:** Fiber source, enzyme combination, and summary statistics of research outcomes of selected research papers evaluating the effects of non-starch polysaccharides-degrading enzymes on average daily gain and feed conversion ratio in broilers.

						ADG in NSPase		ADG in Control		FCR in NSPase		FCR in Control	
S.N.	Study	Days	Fiber Source	Enzyme	Rep (N)	Mean, g	±SEM	Mean, g	±SEM	Mean	±SEM	Mean	±SEM
1	Amerah et al. 2008 [111]	21	w 66.5%	xyl	6	46.5	0.57	47.2	0.57	1.35	0.013	1.41	0.013
2	Amerah et al. 2015 [112]	21	w > 60%, s > 5%, rp 0–6%	xyl + gluc	8	49.6	0.63	49.9	0.63	1.34	0.008	1.35	0.008
3	Gao et al. 2008 [113]	21	w 60%	xyl + gluc + cel + pec	4	37.3	1.57	32.9	1.27	1.58	0.070	1.73	0.065
4	Kiarie et al. 2014 [114]	21	w 60%, wb 9%	xyl + basal phy	6	37.2	0.86	34.8	0.86	1.37	0.023	1.42	0.023
5	La’zaro et al. 2003 [115]	25	r 50%	xyl + gluc	7	38.3	0.99	31.7	0.99	1.66	0.040	1.71	0.040
6	Lee et al. 2020 [116]	20	w 10–20%	xyl	10	36.8	0.42	34.9	0.42	1.47	0.021	1.56	0.021
7	Luo et al. 2009 [117]	21	w 40%	xyl	5	27.9	0.35	27.2	0.35	1.56	0.046	1.71	0.046
8	Mathlouthi et al. 2002 [118]	21	w 40%, b 20%	xyl + gluc	12	38.7	1.21	27.4	1.77	1.50	0.009	1.65	0.030
9	Munyaka et al. 2016 [119]	21	w 42%, b 5%, r 5%, wm 2%	xyl + gluc	7	40.6	0.45	39.3	0.45	1.12	0.040	1.17	0.040
10	Pirgozliev et al. 2015 [120]	21	w 63%	xyl	6	39.4	0.57	38.4	0.57	1.35	0.008	1.35	0.008
11	Selle et al. 2003 [121]	24	w 70%	xyl + phy	8	44.3	0.81	38.4	0.81	1.46	0.021	1.57	0.021
12	Selle et al. 2003 [121]	24	w 70%	xyl	8	44.2	0.81	38.4	0.81	1.48	0.021	1.57	0.021
13	Wang et al. 2005 [109]	21	w 70%	xyl + gluc	6	41.4	0.32	36.9	0.32	1.53	0.013	1.59	0.013
14	Woyengo et al. 2008 [122]	21	w 58%	xyl main effect	8	44.3	0.56	43.7	0.56	1.26	0.008	1.27	0.008
15	Wu et al. 2005 [123]	21	w 66%	xyl	6	38.3	0.39	37.3	0.39	1.34	0.015	1.38	0.015
16	Yang et al. 2008 [124]	21	w 62.4%	xyl	8	56.9	1.26	52.9	1.26	1.65	0.049	1.83	0.049
17	Zhang et al. 2014 [110]	21	w 60%	xyl	6	37.7	0.35	35.6	0.35	1.52	0.030	1.60	0.030

Abbreviations: S.N. serial number; Rep: the number of replicates, NSP: non-starch polysaccharide; ADG: average daily gain; FCR: feed conversion ratio; N: replicate; SEM: pooled standard error of mean; w: wheat; b: barley; r: rye; wb: wheat bran; wm: wheat middlings; rp: rapeseed; s: sunflower; xyl: xylanase; gluc: glucanase; phy: phytase; cel: cellulase; pec: pectinase.

### 6.2. Significance of Exogenous Fiber-Degrading Enzymes during Disease Challenge in Poultry

The efficacy of exogenous enzymes becomes more important during disease challenge conditions in poultry flock when the digestive and immune system of the birds are in a compromised state. The disease condition deteriorates the performance and reduces the efficiency of feed utilization that can further increase the cost of production. Amerah et al. [125] found that in a wheat-based basal diet, xylanase supplementation (2000 U/kg of feed) increased weight gain by 16% and reduced FCR by 6% at day 42 in *Salmonella enterica* serovar Heidelberg (5 × 10^5^ CFU/mL) challenged broilers. In the same study, xylanase supplementation also reduced the *Salmonella*-positive cecal samples from 32.5% in the challenged control to 12.5%. Sun et al. [126] mentioned that the enzyme complex containing xylanase, glucanase, and mannanase as major components supplemented at 500 mg/kg diet decreased *Clostridium perfringens* from 3.66 to 3.48 log CFU/g of ileal digesta, increased body weight by 4%, improved FCR by 3%, increased villus height by 8%, and villus height to crypt depth ratio by 11% in 3-week broilers. Likewise, in a study by Jia et al. [127] on broiler chickens challenged with *Clostridium perfringens*, the supplementation of carbohydrase enzyme complex at 1 kg/ton of feed (supplying 60 U cellulase, 1400 U pectinase, 1200 U xylanase, 800 U glucanase, 500 U mannanase, 30 U galactanase, and other minor enzyme activities per kilogram of diet) reduced the feed conversion ratio by 5–6% in wheat- and flaxseed-containing diets. The mixture of feed enzymes can also be used in combination with direct-fed microbials to improve feed utilization and compensate for the damage and performance loss if occurred due to a coccidial challenge [128]. Jackson et al. [129] supplemented 100 million units of β-mannanase per ton of feed of broilers subjected to necrotic enteritis using a *Eimeria* sp. and *Clostridium perfringens* model. The authors reported that the intestinal lesion score was decreased by 16% on day 14, weight gain was increased by 14%, and FCR was improved by 11% on day 21. In a 39-day broiler study, Choct et al. [130] reported that the inclusion of 2.5 g/kg xylanase enzyme in a wheat-based diet reduced the number of ileal and cecal population of *Clostridium perfringens* to an insignificant level. Bortoluzzi et al. [131] confirmed that the addition of β-mannanase at 400 mg/kg in the diet increased *Lactobacillus* and *Ruminococcaceae* and reduced *Bacteroides* in the ceca of 21-day broilers regardless of the *Eimeria* challenge. Thus, an ideal enzyme or enzyme blend can reduce digesta viscosity, increase available energy, improve nutrient utilization, provide a health benefit, and reduce environmental pollution [132]. Therefore, such exogenous enzymes can prove effective in circumstances where AGPs are not desired. Further, it would be interesting to focus on research to elucidate the host–immune–diet–microbiome interactions to realize the benefits of additives like prebiotic DF and feed enzymes during restricted use of AGPs. Moreover, the supplementation of potent NSPases along with dietary fiber could improve productive performance and gut health of poultry, and thus increase profitability in both healthy and disease-affected flocks.

## 7. Conclusions

Several agricultural co-products are mixed in poultry diet to reduce the cost of feed production. The increase in fiber content of feed from these alternative ingredients limits their inclusion owing to their low digestibility and antinutrient properties. However, there has been further progress in understanding the additional roles of the fiber component of diet in modulating gut microbiome, stimulating immunity, and promoting gut integrity. This advancement in knowledge has made the inclusion of fiber in poultry feeding a matter of further interest and due consideration. Furthermore, the departure of poultry producers from absolute dependence on AGP has also diverted focus towards exploiting the existing properties of feed components and non-AGP additives to achieve similar levels of performance. Unfortunately, no such alternatives have provided a comparable and consistent improvement over AGP. However, the addition of targeted DF components integrated with proper processing techniques and the application of exogenous enzymes can be utilized to maximize the benefits of DF additives while reducing their antinutrient properties, resulting in more efficient and profitable poultry production.

## Data Availability

Not applicable.
